# Inhaled Corticosteroids and Bronchiectasis: Friend or Foe?

**DOI:** 10.3390/jcm12093322

**Published:** 2023-05-07

**Authors:** Miguel Angel Martinez-Garcia

**Affiliations:** 1 Servicio de Neumología, Hospital Universitario y Politécnico la Fe de Valencia, 46026 Valencia, Spain; martinez_miggar@gva.es; Tel.: +34-609865934; 2 CIBERES de Enfermedades Respiratorias, ISCIII, 28222 Madrid, Spain; 3 Pneumology Department, Hospital Universitario y Politécnico la Fe, Avenida Fernando Abril Martorell, 46012 Valencia, Spain

The three most common chronic inflammatory airway diseases are asthma, chronic obstructive pulmonary disease (COPD), and bronchiectasis [1,2,3,4]. From an endotypical point of view, asthma has traditionally been considered a disease with an eosinophilic inflammatory profile [5], while COPD [6] and bronchiectasis have been considered diseases with a neutrophilic inflammatory profiles [7], all of them with increase inflammation markers during exacerbations [8,9,10,11,12]. Nowadays, however, it is known that, in many cases, the three diseases can present mixed bronchial inflammation with a varying percentage of eosinophils and neutrophils, which can be modified depending on the circumstances. Thus, the existence of so-called “neutrophilic asthma”, which is more difficult to manage, is well known [13], while the eosinophilic inflammatory component in COPD (in either the acute or stable phase of the disease) is considered a therapeutic marker of the efficacy and safety of inhaled corticosteroids (ICs) [14]. In the case of bronchiectasis, some authors have recently observed that, along similar lines to COPD, up to 20% of patients (without apparent asthma) may present a high number of eosinophils (at least 300 cells/µL) in peripheral blood as a surrogate marker of the existence of a bronchial eosinophilic inflammation [15,16]. This finding, therefore, immediately opens up a question of great clinical relevance: could ICs be effective in this “eosinophilic bronchiectasis” group?

Prior to addressing this question, there are at least three important issues that should be discussed. Firstly, regardless of the number of peripheral eosinophils, the use of ICs is widespread in patients with bronchiectasis, without any clear indication or recommendation from international guidelines in this regard. Thus, according to the results of the various international bronchiectasis registries, between 39 and 66.7% of these patients have been taking ICs [17]; these figures are complemented, however, by other findings of an increase in the number of exacerbations and hospitalizations in bronchiectasis patients who have used ICs [18]. The cause of this overuse is not clear, but it may be due to a belief that ICs will provide clinical improvement in the most symptomatic or exacerbator patients (as in COPD), or to a tendency to use inhalers that incorporate one or two bronchodilators and one IC in a single device. The second issue is the difficulty in studying the true effect of ICs on bronchiectasis, since the coexistence of asthma would have to be ruled out, but the two diseases sometimes share similar symptoms and lung function alterations. Furthermore, 20% of severe asthmatics also have bronchiectasis [19,20], and the two diseases are so prevalent that a single individual could easily have the misfortune to contract them both separately. Finally, bronchial biopsy samples that would evaluate the true extent of infiltration by eosinophils involve an invasive procedure, so the presence of a high number of eosinophils in peripheral blood is used as an alternative marker of bronchial eosinophilia. However, as in the case of COPD, the correlation with bronchial eosinophilia is only moderate and somewhat non-specific (it can be modified by various factors), and this is a limitation in the interpretation of this value [21].

Once these issues have been considered, it is evident, as the bronchiectasis guidelines indicate, that patients with asthma and bronchiectasis should be treated with ICs, although it might be appropriate to minimize the use of ICs in asthma patients with overlapping bronchiectasis who present a chronic bronchial infection (CBI) by potentially pathogenic microorganisms (PPM), and this may be performed using some alternatives, such as macrolides or leukotriene receptor antagonist therapies [22]. This precaution is derived from the proven immunosuppressive nature of ICs that has been described by various authors [17]. In such cases, the use of a triple therapy of long-acting beta agonist (LABA) + long-acting muscarinic antagonist (LAMA) + low-medium doses of CIs may be more suitable than LABA + high doses of CIs, although this hypothesis would need to be confirmed by future studies. Similarly, the indication of ICs would also be appropriate in those patients with bronchiectasis due to allergic bronchopulmonary aspergillosis (ABPA) [23]. Finally, patients with overlapping bronchiectasis-COPD plus peripheral eosinophilia would also be susceptible to treatment with ICs, although, as in the case of asthma, perhaps their use should be individualized, and the risk-benefit factor should be carefully evaluated if there is also a CBI by PPM [24,25].

The effect of ICs on the group of patients with bronchiectasis in whom the existence of asthma, ABPA, or COPD with peripheral eosinophilia has been reasonably ruled out continues to be a scientific challenge, however. This is a clinical issue of enormous interest that is currently being investigated by several research groups. In fact, some data are already available: some authors have observed the presence of peripheral eosinophilia (>300 eosinophils/µL) in approximately 20% of patients with bronchiectasis [15]. These patients seem to present a greater number of exacerbations, and some small studies seem to suggest that treatment with ICs could benefit them by reducing the number and severity of their exacerbations or improving their quality of life [26,27]. It has also been observed, however, that this relationship between eosinophilia and exacerbations in bronchiectasis follows a non-linear pattern [15,28]. Thus, it seems that the severity and mortality of bronchiectasis is greater, as well as that there is a greater probability of CBI due to PPM in patients with less than 50–100 eosinophils/µL. Therefore, treatment with ICs could even be harmful in such cases. This non-linear relationship could perhaps be explained by the dual nature of eosinophils as pro-inflammatory cells with antimicrobial functions, meaning that both an excess and a deficiency of eosinophils could increase severity or exacerbations in bronchiectasis (although this aspect still remains far from clarified) [5].

In conclusion, although the general premise, supported by the international guidelines on bronchiectasis regarding the avoidance of the use of ICs in these patients (unless there is an association with asthma) is still valid, it is true that the presence of peripheral eosinophilia in these patients may possibly be considered a treatable trait of bronchiectasis in certain cases, as well as that ICs may provide an effective treatment for them. However, these propositions still remain within the realm of hypothesis. Many issues have yet to be resolved, such as the effect of comorbidities, the dose of ICs, the ideal cut-off point to define peripheral eosinophilia in this context as an indicator of which patients to treat, and the potential deleterious effect of ICs on bronchiectasis patients with CBI due to PPM. It is to be hoped that ongoing studies (whether clinical or focused on the pathophysiological pathways and changes in the lung microbiome [29] that govern the relationship between ICs and bronchiectasis) will provide answers to some of these questions in the near future.
